# Energy-Metabolism-Enhancing Probiotics Enhance the Therapeutic Response to a Glucagon-like Peptide-1 Receptor Agonist

**DOI:** 10.3390/nu18071050

**Published:** 2026-03-26

**Authors:** A-Ram Kim, Seong-Gak Jeon, So-Jung Park, Byoung-Kook Kim, Mi-Na Kweon, Myoung Ho Jang, Bo-Gie Yang

**Affiliations:** 1 Research Institute, GI Longevity Inc., Seongnam 13201, Republic of Korea; 2 Mediogen Inc., Jecheon 27159, Republic of Korea; 3 Mucosal Immunology Laboratory, Department of Convergence Medicine, University of Ulsan College of Medicine/Asan Medical Center, Seoul 05505, Republic of Korea; 4 Digestive Diseases Research Center, University of Ulsan College of Medicine/Asan Medical Center, Seoul 05505, Republic of Korea; 5 World Premier International Immunology Frontier Research, Osaka University, Suita 565-0871, Japan; 6 Research Institute, GI Innovation Inc., Seoul 05855, Republic of Korea

**Keywords:** probiotics, *Limosilactobacillus fermentum* GB102, GLP-1 receptor agonist, dulaglutide, obesity, energy expenditure, succinic acid, weight regain

## Abstract

**Background/Objectives**: Glucagon-like peptide-1 receptor agonists (GLP-1RAs) are effective treatments for obesity, but substantial weight regain is common after therapy is discontinued. This study investigated whether probiotic strains with anti-obesity effects could enhance GLP-1RA-induced weight loss and attenuate post-treatment weight rebound. **Methods**: Candidate lactic acid bacteria were screened for anti-obesity efficacy in a high-fat-diet (HFD)-induced obese mouse model, and the selected strain was further characterized using metabolomic profiling of culture supernatants. To examine its interaction with GLP-1RA therapy, obese mice received dulaglutide for 4 weeks and were monitored for 2 weeks after treatment withdrawal, while the probiotic was orally administered for a total of 6 weeks. Body weight, glycemic parameters, and muscle strength were assessed throughout the study. **Results**: *Limosilactobacillus fermentum* GB102 reduced body weight and improved glycemic control in HFD-fed mice. These metabolic benefits were associated with alterations in circulating metabolic hormones, including adipokines, along with attenuated inflammatory responses in adipose tissue. Metabolomic profiling revealed that GB102 produced high levels of succinic acid, a metabolite previously linked to thermogenic activation. This strain increased whole-body energy expenditure in HFD-fed mice, produced glutamine, and showed enhanced conversion of arginine into ornithine and citrulline. When combined with dulaglutide, GB102 enhanced weight loss, preserved muscle strength, and attenuated both weight regain and glycemic rebound following dulaglutide withdrawal. **Conclusions**: These findings suggest that energy-metabolism-enhancing probiotics such as GB102 may enhance the metabolic effects of GLP-1RA therapy and help attenuate weight regain after treatment discontinuation.

## 1. Introduction

Obesity is rapidly increasing worldwide and has become a major public health concern, posing a major threat to population health through its strong association with numerous chronic metabolic diseases [1,2]. It is a major risk factor for type 2 diabetes, cardiovascular complications, and functional decline, contributing substantially to global morbidity and healthcare burden. Glucagon-like peptide-1 receptor agonists (GLP-1RAs) are widely used as effective therapies for obesity and type 2 diabetes, providing clinically meaningful reductions in body weight and improvements in glycemic control [3]. These agents mimic the actions of endogenous GLP-1 by enhancing glucose-dependent insulin secretion, suppressing glucagon release, slowing down gastric emptying, and promoting satiety, thereby contributing to improved metabolic control and reduced energy intake [3]. However, therapeutic responses vary across individuals, and some patients experience limited benefits despite appropriate dosing [4]. Moreover, several clinical studies have shown that the discontinuation of GLP-1RA therapy leads to substantial weight regain, with greater rebound observed in individuals who achieved larger weight reductions [5,6,7]. Glycemic parameters also deteriorate after treatment withdrawal, sometimes worsening beyond pretreatment levels [5,8]. These limitations highlight the need for complementary strategies that enhance the weight-lowering effects of GLP-1RAs and mitigate the rebound weight gain that commonly follows treatment discontinuation. Such approaches, ideally, should be safe, affordable, and sustainable for long-term use, enabling patients to maintain metabolic benefits without added financial or treatment burdens.

Numerous studies have demonstrated that the gut microbiota is closely linked to obesity and related metabolic outcomes [9,10]. An increasing amount of evidence indicates that the gut microbiota acts as a key regulator of host energy balance, muscle function, and inflammatory status [11]. Certain probiotic strains influence these host processes by producing bioactive microbial metabolites derived from carbohydrate and amino acid metabolism [12]. Short-chain fatty acids are among the most extensively studied microbiota-derived metabolites, and they play important roles in appetite regulation and lipid oxidation [11,13]. In addition to SCFAs, organic acids such as succinate have emerged as key metabolic signals that activate thermogenic programs in brown adipose tissue, enhance mitochondrial respiration, and promote the induction of beige adipocytes within white adipose tissue [14,15,16,17,18]. Beyond carbohydrate-derived metabolites, amino acid-related metabolites exert profound effects on muscle and metabolic physiology. Among these, arginine and citrulline are central components of nitric oxide (NO) biosynthesis, a pathway that regulates muscle perfusion, vascular tone, mitochondrial function, and contractile performance [19,20]. Notably, citrulline exhibits superior intestinal absorption and is efficiently converted to arginine in peripheral tissues, resulting in more sustained increases in circulating arginine availability and NO production compared with direct arginine supplementation [21]. Through NO-mediated improvements in blood flow and mitochondrial support, these amino acid-related metabolites suggest that there is a mechanistic link between microbial metabolism and muscle function. Arginine metabolism further gives rise to ornithine, a key precursor for polyamine biosynthesis. Polyamines represent another important class of microbiota-derived metabolites, and they play essential roles in maintaining epithelial barrier integrity, regulating cellular proliferation, modulating immune responses, and supporting metabolic homeostasis [22,23,24,25]. Through these diverse actions, microbial metabolites provide a mechanistic link between gut microbiota activity, thermogenic regulation, muscle function, and systemic metabolic resilience.

In this study, we screened the anti-obesity potential of probiotic candidates in a high-fat-diet (HFD)-induced obese mouse model and identified a candidate strain with distinct metabolic properties. Metabolomic profiling revealed that this strain produced high concentrations of succinic acid and preferentially channeled arginine metabolism toward citrulline and ornithine, metabolites associated with thermogenesis, muscle function, and host nitrogen metabolism. Based on these metabolic features, we investigated whether this probiotic strain could augment the weight-lowering effects of the GLP-1RA dulaglutide, preserve muscle mass during weight reduction, and mitigate the weight regain and glycemic deterioration typically observed after treatment withdrawal. Our findings support the potential use of a microbiota-derived metabolic adjunct approach to possibly improve both the efficacy and durability of GLP-1RA therapy.

## 2. Materials and Methods

### 2.1. Preparation of Lactic Acid Bacteria

The probiotic strains used in this study were obtained from diverse human- or food-derived sources. Several candidate strains were originally isolated from the vaginal microbiota of healthy Korean women, a well-recognized reservoir of lactic acid bacteria with probiotic potential and a common source of human-derived *Lactobacillus* strains [26]. These included *Limosilactobacillus fermentum* GB102 (=MG4261), MG4258, and *Lactiplantibacillus plantarum* GB104 (=MG4270) [27]. *L. plantarum* MG5120 was isolated from shellfish [27], whereas *Lactobacillus helveticus* GB201 (=MG585) was isolated from a fermented milk product [28]. *L. fermentum* KCTC3112 was obtained from the Korean Collection for Type Cultures (KCTC). For the animal experiments, the strains were cultured and lyophilized using Mediogen (Jecheon, Republic of Korea). For the metabolomic analyses, the strains were cultured in de Man–Rogosa–Sharpe (MRS) medium for 6 h. To assess succinic acid production, cultures were incubated for either 3 or 6 h in GIL medium alone, GIL medium supplemented with 0.4% (*w*/*v*) citrate, or GIL medium supplemented with 0.4% (*w*/*v*) tartrate. The GIL medium consisted of yeast peptone FNI 800, baker’s yeast extract, dextrose, sodium sulfate, ammonium sulfate, dibasic potassium phosphate, magnesium sulfate, manganese sulfate, iron sulfate, zinc sulfate, and Tween 80. For in vitro assays, bacterial cultures were grown under anaerobic conditions using a Whitley A45 anaerobic workstation (Don Whitley Scientific, Bingley, UK) under a mixed gas atmosphere (N_2_/H_2_/CO_2_, 90:5:5).

### 2.2. Animal Experiments

All animal experiments were conducted at the animal facilities of the Seoul National University Institute of Systems Immunology (SNU-ISI; Hongcheon, Republic of Korea) and GI-Biome Inc. (Seongnam, Republic of Korea). The experimental procedures were approved by the Institutional Animal Care and Use Committee (IACUC) of SNU-ISI (SNU-190212, 12 February 2019) and GI-Biome (GIB-21-04-009, 27 April 2021; GIB-21-11-006, 4 November 2021; GIB-21-05-012, 25 May 2021; GIB-22-03-002, 14 March 2022; GIB-22-04-011, 19 April 2022; GIB-24-05-003, 10 May 2024). Five-week-old male C57BL/6 mice were purchased from Orient Bio (Seongnam, Republic of Korea) and acclimatized for one week before experimentation. Mice were maintained under specific-pathogen-free (SPF) conditions in temperature-controlled rooms with a 12 h light/dark cycle.

Mice in the high-fat diet (HFD) group were fed a 60 kcal% fat diet (D12492; Research Diets, New Brunswick, NJ, USA), whereas mice in the normal chow diet (NCD) group received a standard chow diet (Teklad Global 18% Protein Rodent Diet, 2018S; Inotiv, Lafayette, IN, USA). Probiotic strains were administered orally once a day starting at the onset of HFD feeding. Lyophilized bacterial powders were reconstituted in phosphate-buffered saline (PBS) and administered at doses of 1 × 10^10^ or 5 × 10^9^ CFU in a total volume of 200 μL per mouse. Body weight was recorded weekly. Whole-body composition was assessed using time-domain nuclear magnetic resonance (TD-NMR) with an LF50 analyzer (Bruker, Billerica, MA, USA). In a separate experiment, obesity was induced by prolonged HFD feeding before the initiation of dulaglutide and GB102 treatment. Dulaglutide (1 nmol/kg) was administered subcutaneously every other day for a total of 4 weeks. GB102 was administered orally for a total of 6 weeks, consisting of 4 weeks of co-administration with dulaglutide followed by 2 additional weeks of GB102 alone. In obese mice with an average body weight of ~42 g, GB102 was administered at 1 × 10^10^ CFU/200 μL per mouse, whereas mice with a slightly lower body weight (≤40 g) received 5 × 10^9^ CFU/200 μL per mouse. The sample size for each experimental group was determined in accordance with the ethical principles of the 3Rs (Replacement, Reduction, and Refinement) to minimize animal use while maintaining statistical validity.

### 2.3. Glucose Tolerance Test (GTT)

The GTT was performed after 16 h of fasting. Fasting blood glucose was measured from the tail vein using an Auto-check Plus glucose meter (GM01RAA; i-SANS, Seoul, Republic of Korea). Mice were injected intraperitoneally with glucose (1 g/kg body weight), and blood glucose levels were recorded at 15, 30, 60, and 120 min. The area under the curve (AUC) was calculated using GraphPad Prism version 9.5.1 (GraphPad Software, La Jolla, CA, USA).

### 2.4. Insulin Tolerance Test (ITT)

The ITT was performed after 4.5 h of fasting. Baseline blood glucose was measured before an intraperitoneal injection of insulin (1 U/kg body weight). Blood glucose levels were measured at 15, 30, 60, and 120 min using the same glucose meter. AUC values were calculated using GraphPad Prism version 9.5.1.

### 2.5. Measurement of Circulating Metabolic Hormones and Adipokines

The serum levels of metabolic hormones and adipokines were quantified using the Bio-plex Pro Mouse Diabetes 8-plex assay kit (Bio-Rad, Hercules, CA, USA) on a Bio-Plex 200 system (Bio-Rad). All procedures were performed according to the manufacturer’s instructions.

### 2.6. Histological Analysis

Liver and adipose tissue samples were fixed in 10% neutral buffered formalin (NBF), embedded in paraffin, sectioned, and stained with hematoxylin and eosin (H&E). All histological procedures were performed at the Seoul National University Institute of Systems Immunology.

### 2.7. Isolation of Immune Cells from Adipose Tissue

Immune cells were isolated as previously described [29]. Briefly, epididymal adipose tissues were minced and digested in RPMI 1640 medium containing 400 U/mL of collagenase D (Roche, Mannheim, Germany), 10 μg/mL of DNase I (Roche), 1 mM of sodium pyruvate, and 1 mM non-essential amino acids (NEAAs) at 37 °C for 45 min. The enzymatic reaction was stopped by adding EDTA to a final concentration of 10 mM. The digested tissue was passed through a 40 μm cell strainer, and immune cells were enriched using a 40/75% Percoll (Cytiva, Marlborough, MA, USA) gradient.

### 2.8. Flow Cytometric Analysis of Immune Cells

Fluorophore-conjugated monoclonal antibodies were used against the following markers: MHCII (M5/114.15.2), F4/80 (BM8), CD11b (M1/70), CD11c (HL3), CD206 (C068C2), TCRβ (H57-597), CD4 (RM4-5), and Foxp3 (FJK-16s). Fc receptors were blocked using anti-CD16/CD32 (TruStain FcX; BioLegend, San Diego, CA, USA). Macrophages were identified based on the expression of CD11b, F4/80, CD11c, CD206, and MHCII. For regulatory T cell (Treg) staining, cells were surface-stained for TCRβ and CD4, followed by intracellular staining for Foxp3 using Foxp3/Transcription Factor Staining Buffer Set (eBioscience, San Diego, CA, USA). Samples were acquired using an LSRFortessa flow cytometer (BD Biosciences, San Jose, CA, USA), and data were analyzed using FlowJo software (version 10.7.1; Tree Star, San Carlos, CA, USA).

### 2.9. Metabolomic Profiles of Bacterial Culture Supernatants

Metabolomic profiling was performed by Human Metabolome Technologies (HMT; Tsuruoka, Japan) using capillary electrophoresis time-of-flight mass spectrometry (CE-TOFMS). Bacterial culture supernatants were collected and filtered through a 0.2 μm membrane filter. Filtrates were then mixed with 20 μL of Milli-Q water containing internal standards at a final concentration of 1000 μM. Cationic and anionic metabolites were analyzed using an Agilent CE-TOFMS system equipped with a fused-silica capillary (50 μm i.d., 80 cm). The conditions for run buffers, ionization modes, injection parameters, scan ranges, and dilution factors were set according to HMT’s standard protocol. Peaks were extracted using MasterHands software (version 2.18.0.1; Keio University, Japan), and relative peak areas were calculated via normalization to internal standards. Metabolites were identified based on migration time (±0.5 min) and *m*/*z* (±10 ppm) using HMT’s standard and Known–Unknown libraries. Absolute quantification of target metabolites was performed using calibration based on single-point standards (100 μM). Metabolite concentrations were calculated by normalizing each metabolite peak to its corresponding internal standard.

### 2.10. Analysis of Succinic Acid Using Gas Chromatography–Mass Spectrometry (GC-MS)

Bacterial culture supernatants (200 μL) were extracted with 800 μL of methanol and homogenized using a mixer mill (MM400; Retsch, Haan, Germany). After centrifugation, samples were filtered through a 0.2 μm PTFE membrane filter, dried using a vacuum concentrator (HyperVac-Max; Labex, Namyanju, Republic of Korea), and resuspended in methanol to a final concentration of 20 mg/mL. Derivatization was performed with methoxyamine hydrochloride (20 mg/mL in pyridine; 30 °C, 90 min), followed by *N*-methyl-*N*-trimethylsilyl trifluoroacetamide (MSTFA) treatment (37 °C, 30 min). Samples were analyzed on a Clarus GC system (PerkinElmer, Waltham, MA, USA) equipped with a PAL3 autosampler and a YL6900 mass spectrometer (YL Instrument, Anyang, Republic of Korea) using a DB-5MS column. Mass spectra were acquired in EI mode (70 eV; *m*/*z* 50–600). Succinic acid abundance was quantified using Clarity software (version 8.1) based on peak identities confirmed with authentic standards.

### 2.11. Energy Expenditure and Locomotor Activity Analysis

Energy expenditure was measured using the Comprehensive Lab Animal Monitoring System (CLAMS; Columbus Instruments, Columbus, OH, USA). Mice were housed individually in metabolic chambers, and measurements were recorded continuously for 48 h under a 12 h light/dark cycle (lights on at 09:00) at 22 °C. Oxygen consumption (VO_2_) and carbon dioxide production (VCO_2_) were recorded hourly. Locomotor activity was simultaneously monitored using infrared beam breaks within the CLAMS system. Energy expenditure (kcal/h/kg) was calculated from VO_2_ and VCO_2_ data using Oxymax for Windows software (version 5.57; Columbus Instruments) and normalized to body weight. AUC values for energy expenditure and locomotor activity were calculated using GraphPad Prism version 9.5.1.

### 2.12. Statistical Analysis

All statistical analyses were performed using GraphPad Prism version 9.5.1. Data from in vitro experiments are presented as mean ± standard deviation (SD), whereas data from in vivo experiments are presented as mean ± standard error of the mean (SEM). Student’s two-tailed unpaired *t*-test was used to make comparisons between the two groups, whereas a one-way analysis of variance (ANOVA) was used to make comparisons between more than two groups, followed by Tukey’s post hoc test for multiple comparisons. The sample size (*n*) for each experiment is indicated in the corresponding figure legends. A *p*-value < 0.05 was considered statistically significant. Statistical significance is indicated as follows: * *p* < 0.05, ** *p* < 0.01, *** *p* < 0.001, and **** *p* < 0.0001.

## 3. Results

### 3.1. L. fermentum GB102 Exhibits Anti-Obesity and Metabolic Benefits in HFD-Induced Obese Mice

**Figure 1:**
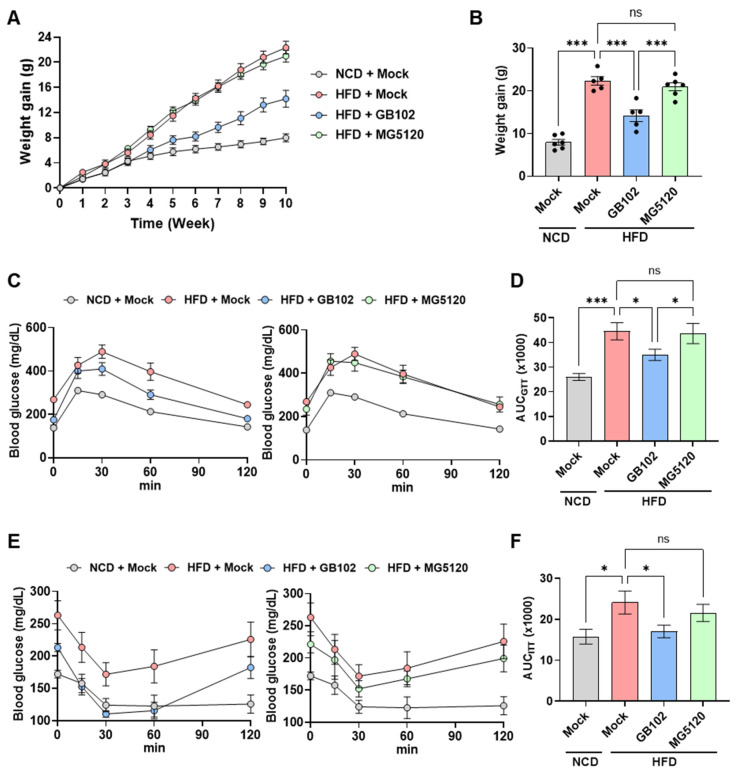
Anti-obesity and metabolic benefits of GB102 in HFD-induced obese mice. Effects of *L. fermentum* GB102 and *L. plantarum* MG5120 (5 × 10^9^ CFU/mouse/day, oral administration) were compared in a high-fat-diet (HFD)-induced obese mouse model. (**A**) Weekly body weight changes. (**B**) Body weight gain at HFD week 10. (**C**,**D**) Glucose tolerance test (GTT) performed at HFD week 10: (**C**) glucose curves and (**D**) corresponding area under the curve (AUC). (**E**,**F**) Insulin tolerance test (ITT) performed at HFD week 11: (**E**) glucose curves and (**F**) corresponding AUC. Data are presented as mean ± SEM (*n* = 5–6 per group). Endpoint data (**B**,**D**,**F**) were analyzed using one-way ANOVA followed by Tukey’s post hoc test. * *p* < 0.05; *** *p* < 0.001; ns, not significant.

To identify probiotic strains with anti-obesity activity, we screened 26 lactic acid bacterial strains in mice fed a 60% high-fat diet (HFD) and identified *L. fermentum* GB102 as a strain that suppressed body weight gain. Among the screened strains, *L. plantarum* MG5120, which did not exhibit significant anti-obesity effects, was selected as a comparator strain for subsequent analyses. Compared with MG5120, GB102 significantly attenuated HFD-induced weight gain (Figure 1A,B). In addition, GB102 significantly improved glucose homeostasis, as evidenced by reduced blood glucose levels during the glucose tolerance test (GTT; Figure 1C,D) and improved insulin sensitivity in the insulin tolerance test (ITT; Figure 1E,F), whereas MG5120 did not show comparable effects.

**Figure 2:**
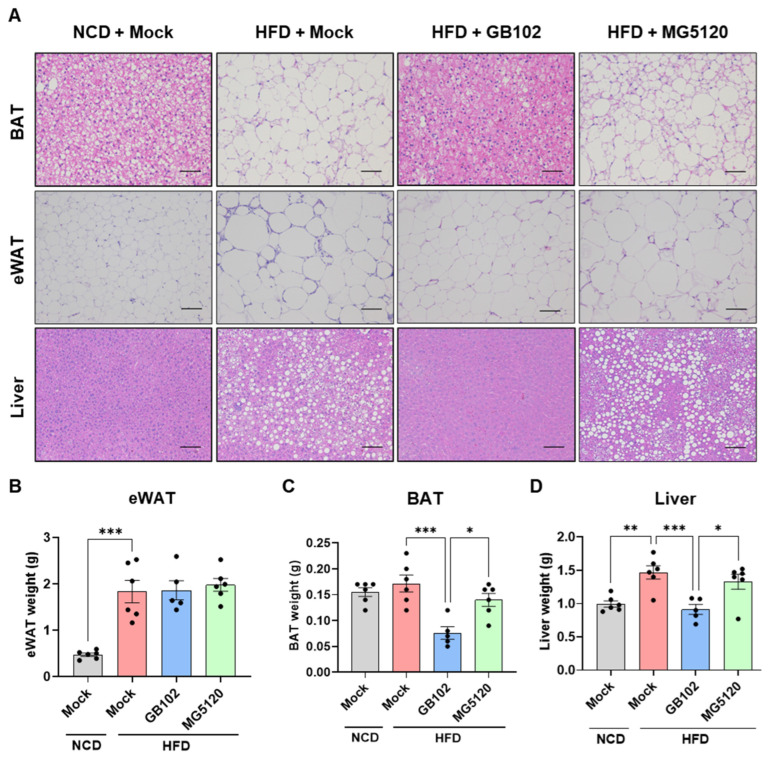
Effects of GB102 on lipid accumulation and tissue mass in adipose and liver tissues. (**A**–**D**) Lipid accumulation and tissue weights after 14 weeks of administration of GB102 or MG5120 (5 × 10^9^ CFU/mouse/day) in HFD-fed mice. Brown adipose tissue (BAT) and epididymal white adipose tissue (eWAT) were analyzed as representative adipose depots. (**A**) Representative hematoxylin and eosin (H&E)-stained sections of BAT (scale bar = 50 μm), eWAT (scale bar = 100 μm), and liver (scale bar = 100 μm) tissues. (**B**–**D**) Weights of adipose tissue depots and liver. Data are presented as mean ± SEM (*n* = 5–6 per group). Statistical comparisons among groups were performed using one-way ANOVA followed by Tukey’s post hoc test. * *p* < 0.05; ** *p* < 0.01; *** *p* < 0.001.

Histological analysis of paraffin-embedded adipose and liver tissues revealed that GB102, but not MG5120, suppressed HFD-induced lipid accumulation in both epididymal white adipose tissue (eWAT) and brown adipose tissue (BAT) (Figure 2A). In eWAT, GB102 markedly reduced adipocyte hypertrophy, indicating improved lipid handling at the cellular level; however, this reduction in adipocyte size did not result in a significant change in total eWAT mass (Figure 2B). In contrast, GB102 significantly reduced both BAT and liver weights (Figure 2C,D). Collectively, these findings indicate that *L. fermentum* GB102 confers anti-obesity effects and metabolic advantages in HFD-fed obese mice.

### 3.2. L. fermentum GB102 Modulates Metabolic Hormones and Adipokines and Attenuates Adipose Tissue Inflammation in HFD-Fed Mice

To determine whether the anti-obesity activity of GB102 is associated with systemic metabolic regulation, circulating metabolic hormones and adipokines were measured using the Bio-Plex Pro Mouse Diabetes 8-plex assay. Insulin and glucagon, key hormones regulating glucose homeostasis, were significantly increased and decreased, respectively, in HFD-fed mice; however, these changes were not significantly altered by GB102 (Figure S1A). Notably, despite the fact that no significant changes in individual hormone levels were detected, the elevated insulin/glucagon ratio induced by HFD was significantly reduced by GB102, whereas MG5120 exerted no detectable effect (Figure S1A). In addition to systemic metabolic hormones, gut-derived hormones were also altered by HFD. Glucagon-like peptide-1 (GLP-1) and ghrelin levels were decreased, whereas glucose-dependent insulinotropic polypeptide (GIP) levels were increased (Figure S1B). GB102 did not significantly restore circulating GLP-1 levels, although a modest upward trend was observed with substantial inter-individual variability (Figure S1B). This lack of a significant increase is consistent with previous in vitro findings showing that GB102 does not directly stimulate GLP-1 secretion [29]. In contrast, GB102 effectively normalized both ghrelin and GIP, while MG5120 influenced only GIP in a manner similar to GB102 (Figure S1B). Among adipose tissue-derived factors, leptin and the inflammatory adipokines resistin and plasminogen activator inhibitor-1 (PAI-1) were significantly elevated in HFD-fed mice (Figure S1C). GB102 showed a trend toward reduction in HFD-induced leptin levels, although this effect did not reach statistical significance. In contrast, GB102 significantly suppressed the HFD-associated elevations in resistin and PAI-1 (Figure S1C). In comparison, MG5120 did not significantly affect HFD-induced leptin levels, but it did significantly reduce resistin levels. Notably, MG5120 further increased PAI-1 levels compared with HFD-fed controls, indicating a divergent effect on this adipokine (Figure S1C).

Because GB102 reduced inflammatory adipokines such as resistin and PAI-1, we next examined whether it also modulated inflammatory immune cell populations within epididymal adipose tissue. HFD increased pro-inflammatory M1 macrophages and reduced anti-inflammatory M2 macrophages, whereas regulatory T cells (Tregs) were not significantly altered (Figure S2A–D). GB102 significantly reduced M1 macrophages and increased Treg abundance (Figure S2A,C,D). Although M2 macrophages showed a modest upward trend with GB102 treatment, this change did not reach statistical significance (Figure S2B,D). In contrast, MG5120 did not produce comparable effects on any of these immune cell populations (Figure S2A–D). Taken together, these findings indicate that GB102 mitigates HFD-induced alterations in metabolic hormones and inflammatory adipokines and alleviates adipose tissue inflammation by reshaping key immune cell subsets.

### 3.3. L. fermentum GB102 Exhibits Distinct Metabolic Features Including High Succinic Acid Production and Enhanced Arginine and Glutamine Metabolism

**Figure 3:**
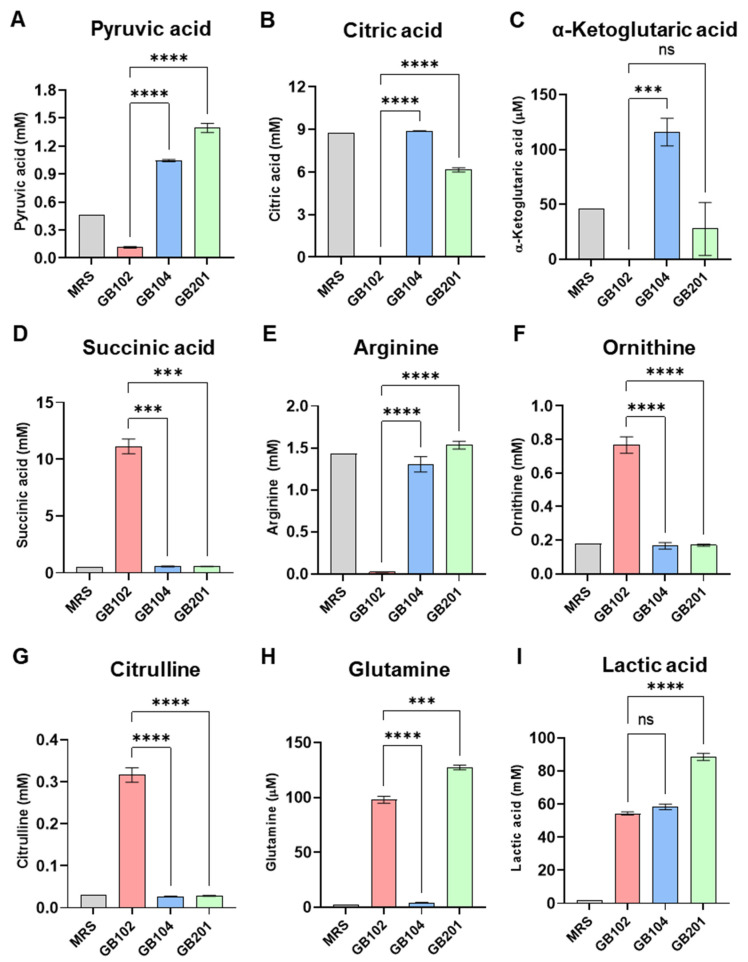
Metabolomic profiling of GB102 culture supernatants. Metabolomic profiling of bacterial culture supernatants collected after 6 h of incubation was performed using capillary electrophoresis time-of-flight mass spectrometry (CE-TOFMS). *Lactiplantibacillus plantarum* GB104 *and Lactobacillus helveticus* GB201 were included as comparator strains. The MRS medium control was analyzed once (*n* = 1), whereas bacterial culture supernatants were analyzed in triplicate (*n* = 3). (**A**–**C**) Tricarboxylic acid (TCA) cycle-related metabolites (pyruvic acid, citric acid, and α-ketoglutaric acid). (**D**–**G**) Urea cycle-related amino acids (arginine, ornithine, and citrulline). (**H**) Glutamine. (**I**) Lactic acid. Data are presented as mean ± SD. Statistical comparisons among bacterial strains were performed using one-way ANOVA followed by Tukey’s post hoc test. *** *p* < 0.001; **** *p* < 0.0001; ns, not significant.

Because the anti-obesity effects of GB102 might be associated with metabolites produced by the strain, metabolomic profiling of bacterial culture supernatants was performed using CE-TOFMS. *L. plantarum* GB104 and *L. helveticus* GB201 served as comparator strains. GB104 was selected as a representative strain with previously reported anti-obesity activity [29], whereas GB201 was included as a comparator strain without known anti-obesity activity but with potential anti-inflammatory properties observed in preliminary studies. CE-TOFMS detected 296 metabolites, of which 74 were quantified. Selected metabolites showing notable differences among strains are presented in Figure 3, whereas the complete set of quantified metabolites is provided in Table S1. Several metabolites exhibited strain-specific patterns in GB102, including TCA cycle-related metabolites (pyruvic acid, citric acid, α-ketoglutaric acid, and succinic acid) and urea cycle amino acids (arginine, ornithine, and citrulline) (Figure 3A–G).

Compared with GB104 and GB201, GB102 showed markedly lower levels of pyruvic acid, citric acid, and α-ketoglutaric acid while markedly increasing succinic acid levels in the culture supernatant (Figure 3A–D). GB102 also showed pronounced depletion of arginine, accompanied by increased levels of its downstream metabolites, ornithine and citrulline (Figure 3E–G). Consistent with this pattern, spermidine levels appeared higher in GB102 compared with the comparator strains, whereas putrescine levels did not show notable differences, and spermine levels were higher in GB201 (Table S1). In addition to these metabolites, GB102 produced higher levels of glutamine compared with GB104, whereas glutamine levels were highest in GB201 among the tested strains (Figure 3H). All strains produced lactic acid, as expected for lactic acid bacteria, with GB201 exhibiting the highest levels (Figure 3I).

**Figure 4:**
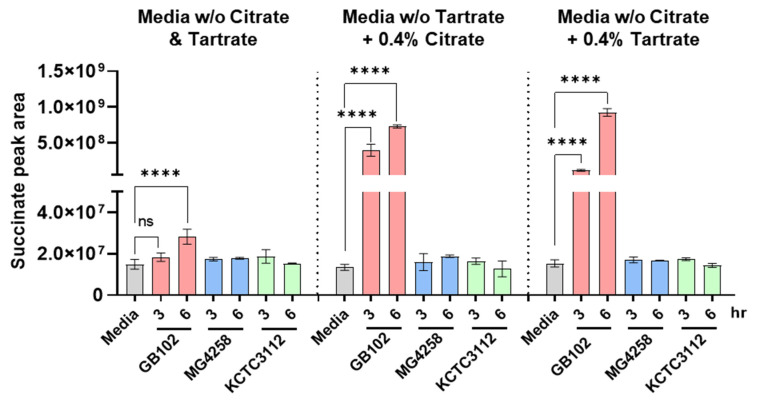
Succinic acid production enhanced by GB102. Relative levels of succinic acid in culture supernatants of *L. fermentum* GB102, MG4258, and KCTC3112 grown in GIL medium with or without citrate or tartrate were measured using gas chromatography–mass spectrometry (GC-MS). Each group was analyzed in triplicate (*n* = 3 per group). Data are presented as mean ± SD. Statistical comparisons among groups were performed using one-way ANOVA followed by Tukey’s post hoc test. **** *p* < 0.0001; ns, not significant.

To further assess whether GB102 preferentially utilizes organic acids for succinic acid production, the strain was cultured in a base medium lacking citrate or tartrate and then supplemented with either citrate or tartrate, and succinic acid levels were measured over time. *L. fermentum* MG4258 and KCTC3112, which belong to the same species as GB102, were included as comparator strains to assess whether the observed metabolic phenotype was strain-specific rather than species-wide. GB102 produced substantially higher amounts of succinic acid in citrate-supplemented medium than in citrate-free medium at both 3 h and 6 h (Figure 4). Although succinic acid levels also increased in the absence of citrate, they remained markedly lower than under citrate-supplemented conditions. Notably, GB102 produced similarly elevated succinic acid levels when cultured in tartrate-supplemented medium (Figure 4). In contrast, MG4258 and KCTC3112, which also belong to the *L. fermentum* species, produced little or no succinic acid under the same conditions.

Collectively, these results indicate that GB102 exhibits a distinct metabolic profile characterized by high-level succinate production from citrate or tartrate and active conversion of arginine into ornithine and citrulline, together with characteristic patterns of amino acid metabolism including glutamine production. These strain-specific metabolic pathways may contribute to the anti-obesity activity of GB102.

### 3.4. L. fermentum GB102 Increases Energy Expenditure and Attenuates Diet-Induced Muscle Loss

**Figure 5:**
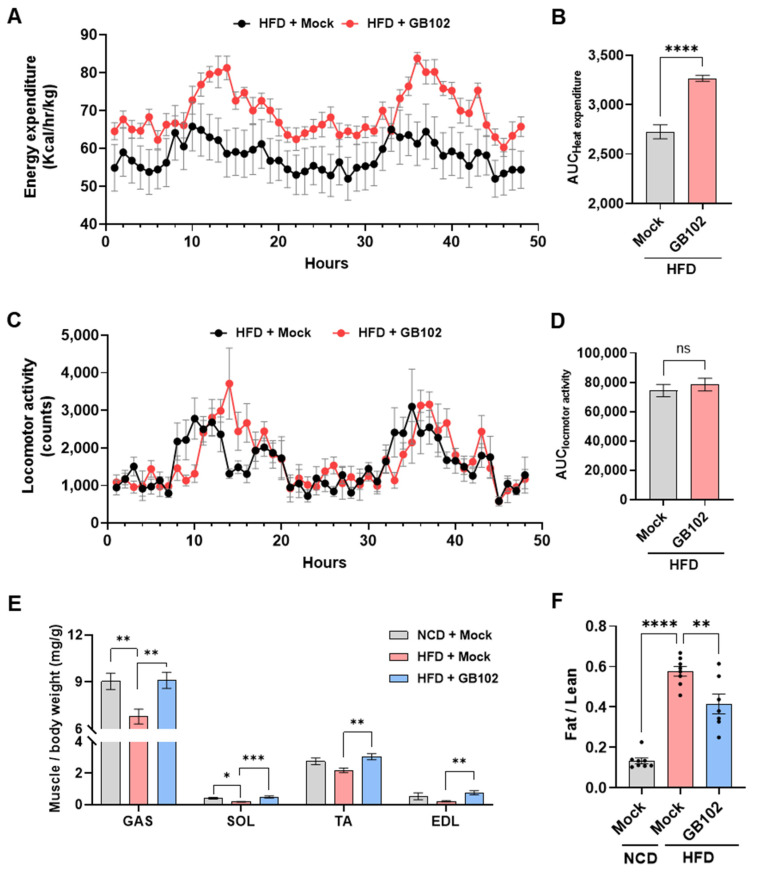
Effects of GB102 on energy expenditure and muscle mass in HFD-fed mice. (**A**,**B**) Energy expenditure in HFD-fed mice following 8 weeks of administration of GB102 (5 × 10^9^ CFU/mouse/day) or PBS. (**A**) Energy expenditure over time. (**B**) Corresponding area under the curve (AUC) (*n* = 6 per group). (**C**,**D**) Locomotor activity following 8 weeks of administration of GB102 or PBS. (**C**) Locomotor activity over time. (**D**) Corresponding AUC (*n* = 6 per group). (**E**,**F**) Body composition and muscle mass in HFD-fed mice following GB102 or PBS administration. (**E**) Muscle mass normalized to body weight at week 9 for gastrocnemius (GAS), soleus (SOL), tibialis anterior (TA), and extensor digitorum longus (EDL). (**F**) Fat-to-lean ratio at week 8 (*n* = 7–10 per group). Data are presented as mean ± SEM. AUC data (**B**,**D**) were analyzed using Student’s two-tailed unpaired *t*-test. Endpoint data (**E**,**F**) were analyzed using one-way ANOVA followed by Tukey’s post hoc test. * *p* < 0.05; ** *p* < 0.01; *** *p* < 0.001; **** *p* < 0.0001; ns, not significant.

Because succinic acid has been reported to promote thermogenic pathways [14,15], and GB102 produced this metabolite in markedly higher amounts than related strains (Figure 3D), we first examined whether GB102 supplementation affected whole-body energy metabolism in HFD-fed mice. In CLAMS analysis, oxygen consumption (VO_2_) and carbon dioxide production (VCO_2_) were monitored hourly for 48 h, and energy expenditure was calculated based on these gas-exchange parameters. GB102-treated mice exhibited significantly higher energy expenditure than mock-treated controls, despite exhibiting comparable locomotor activity (Figure 5A–D).

We next evaluated muscle-related outcomes in light of the distinct amino acid metabolic profile of GB102. Both arginine and citrulline play roles in muscle physiology; however, citrulline exhibits substantially higher intestinal bioavailability than arginine. Because GB102 efficiently converts arginine into citrulline, we assessed whether this strain mitigated HFD-induced muscle loss. In addition, metabolomic analysis further showed that GB102 produced glutamine, an amino acid known to support muscle metabolism. In line with these observations, HFD feeding markedly reduced muscle mass relative to body weight, whereas this decline was prevented in GB102-treated mice. GB102 also significantly lowered the HFD-induced increase in the fat-to-lean ratio (Figure 5E,F).

Taken together, these findings indicate that GB102 increases energy expenditure and preserves muscle mass in HFD-fed mice, supporting the potential for metabolite-associated mechanisms to play a role in underlying its anti-obesity activity.

### 3.5. L. fermentum GB102 Enhances the Efficacy of the GLP-1 Analog Dulaglutide in Obese Mice

**Figure 6:**
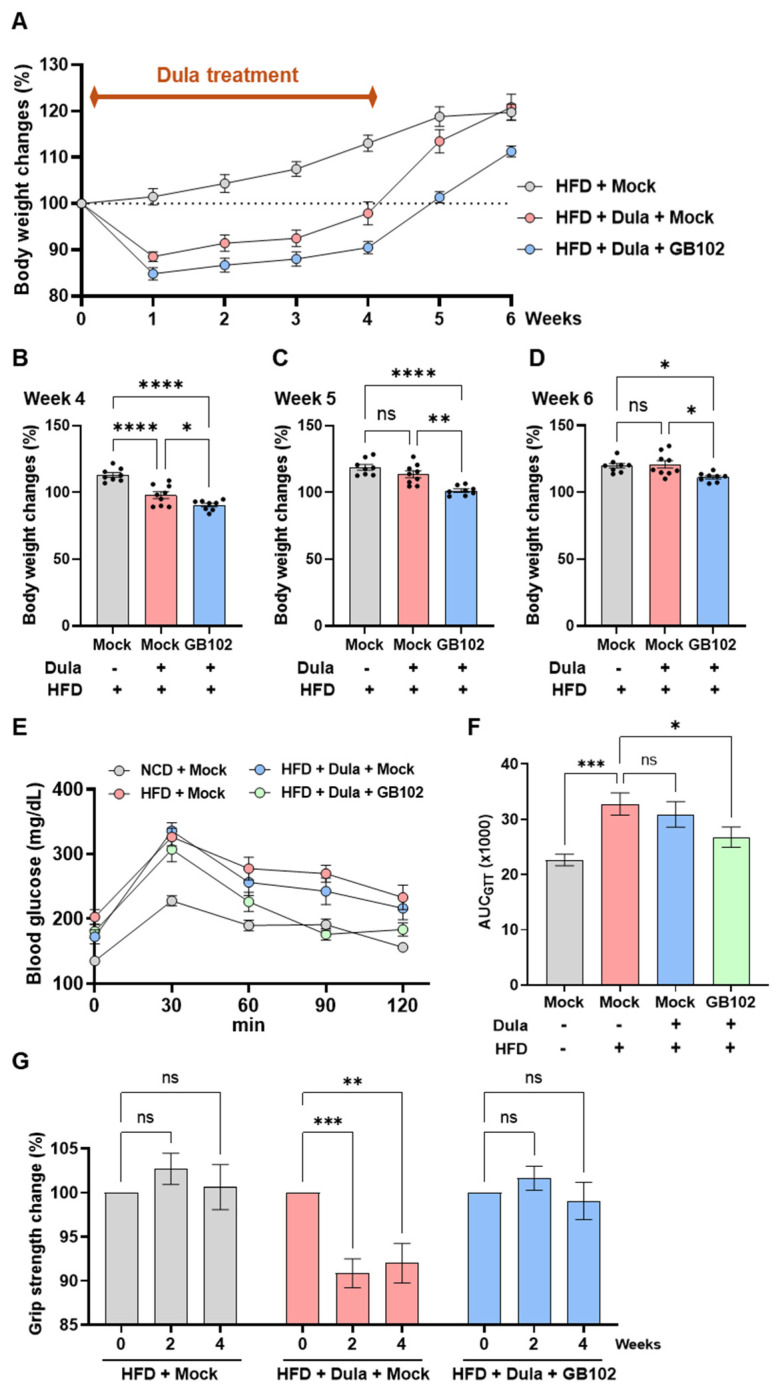
Effects of GB102 on dulaglutide efficacy and post-treatment outcomes in obese mice. (**A**–**D**) Body weight changes in obese mice treated with dulaglutide (1 nmol/kg, s.c., every other day for 4 weeks) with or without GB102 (1 × 10^10^ CFU/mouse, oral, 6 weeks). (**A**) Weekly body weight changes. (**B**) Body weight change at week 4. (**C**) Body weight change 1 week after cessation of dulaglutide (week 5). (**D**) Body weight change 2 weeks after cessation (week 6) (*n* = 8–9 per group). (**E**,**F**) Glucose tolerance test (GTT) performed at week 6 in obese mice treated with dulaglutide (1 nmol/kg, 4 weeks) with or without GB102 (5 × 10^9^ CFU/mouse, 6 weeks). (**E**) GTT glucose curves. (**F**) Area under the curve (AUC) (*n* = 10 per group). (**G**) Grip strength after 4 weeks of treatment with dulaglutide (1 nmol/kg) with or without GB102 (5 × 10^9^ CFU/mouse) (*n* = 10 per group). Data are presented as mean ± SEM. Statistical comparisons for endpoint data (**B**–**D**,**F**,**G**) were performed using one-way ANOVA followed by Tukey’s post hoc test. * *p* < 0.05; ** *p* < 0.01; *** *p* < 0.001; **** *p* < 0.0001; ns, not significant.

To evaluate whether GB102 augments the metabolic benefits of the GLP-1 analog dulaglutide and prevents weight regain after treatment cessation, HFD-induced obese mice were divided into three groups. The first group received dulaglutide alone for 4 weeks, the second group received dulaglutide combined with GB102 for 4 weeks followed by GB102 alone for an additional 2 weeks (total GB102 exposure of 6 weeks), and the control group received no treatment. All mice remained on an HFD throughout the study. As expected, dulaglutide monotherapy reduced body weight during the 4-week treatment period but was followed by rapid weight regain, resulting in body weights that ultimately approached those of untreated obese controls (Figure 6A). In contrast, mice treated with dulaglutide and GB102 exhibited significantly greater weight loss during the combination phase and substantially attenuated weight regain during the subsequent GB102-only phase (Figure 6A–D).

Glucose tolerance was evaluated 2 weeks after the cessation of dulaglutide. Dulaglutide monotherapy did not show a sustained benefit, as glucose excursions during the GTT resembled those of untreated obese mice (Figure 6E,F). In contrast, mice that received the combination regimen followed by GB102 alone displayed significantly reduced glucose excursions, indicating improved glycemic control (Figure 6E,F). Because alterations in body weight can affect muscle performance, we also examined grip strength. Dulaglutide monotherapy resulted in a significant decline in grip strength after 4 weeks of treatment, whereas this reduction was not observed in mice receiving the combined dulaglutide and GB102 regimen (Figure 6G). These findings indicate that GB102 preserves muscle function during dulaglutide therapy.

Together, these data indicate that GB102 potentiates the weight- and glucose-lowering effects of dulaglutide, mitigates post-treatment rebound in both body weight and glycemia, and additionally prevents the decline in muscle strength associated with dulaglutide monotherapy.

## 4. Discussion

In this study, we identified *L. fermentum* GB102 as a probiotic strain associated with multiple beneficial metabolic outcomes in a high-fat-diet (HFD)-induced obese mouse model. GB102 supplementation was associated with increased whole-body energy expenditure, preservation of skeletal muscle mass under HFD conditions, and improvements in metabolic parameters, including body weight and glycemic control. In addition, metabolomic profiling revealed that GB102 exhibits a distinct metabolic signature characterized by high succinic acid production, enhanced conversion of arginine into citrulline and ornithine, and increased glutamine levels. These features provide a basis for interpreting the observed physiological effects, although causal relationships were not directly established in the present study.

Succinic acid has been reported as a microbiota-derived metabolite capable of modulating host energy metabolism, including activation of thermogenic pathways in brown adipose tissue [11,17]. In the present study, GB102 produced high levels of succinic acid and was associated with increased energy expenditure without changes in locomotor activity. In parallel, a reduction in brown adipose tissue mass was observed in GB102-treated mice. These findings are consistent with the possibility that GB102 influences thermogenic or mitochondrial metabolic processes; however, direct measurements of thermogenic gene expression or mitochondrial activity were not performed, and therefore, mechanistic conclusions should be interpreted with caution.

GB102 also exhibited enhanced conversion of arginine into citrulline, a metabolite with higher intestinal bioavailability and the capacity to contribute to systemic arginine pools [21]. Arginine and citrulline are involved in nitric oxide (NO) biosynthesis, which plays roles in vascular function, muscle perfusion, and mitochondrial activity [19,20]. Although circulating levels of these metabolites or NO were not measured in this study, the observed increase in citrulline production is consistent with a potential contribution to muscle-related outcomes. In this context, the attenuation of HFD-induced muscle loss and the preservation of muscle strength observed in GB102-treated mice may be partially associated with these metabolic features, although direct causal links remain to be established.

Metabolomic analysis further indicated that GB102 produced glutamine, an amino acid known to support muscle metabolism and metabolic homeostasis. Glutamine has been reported to influence protein turnover, glucose metabolism, and cellular stress responses in skeletal muscle [30,31]. Therefore, the presence of increased glutamine in GB102 culture supernatants may be associated with the preservation of muscle-related outcomes and improved metabolic parameters observed in this study. However, as systemic glutamine levels were not assessed in this study, the physiological contribution of this pathway requires further investigation.

In addition, GB102 increased ornithine levels, suggesting the engagement of pathways related to polyamine biosynthesis. Ornithine serves as a precursor for polyamines such as putrescine, spermidine, and spermine, which have been implicated in metabolic regulation and cellular homeostasis [25]. In the present dataset, spermidine levels were higher in the GB102 culture supernatant compared with comparator strains, whereas putrescine levels did not show marked differences, and spermine levels were higher in GB201. Although these observations suggest a potential association between GB102-derived metabolism and polyamine-related pathways, polyamine levels in host tissues were not measured, and thus, this interpretation remains speculative.

GLP-1 receptor agonists (GLP-1 RAs) are among the most effective pharmacological treatments for obesity; however, variability in treatment response and substantial weight regain after treatment discontinuation remain important clinical challenges [4,5,6,7,8]. In the present study, co-administration of GB102 with the GLP-1RA dulaglutide was associated with enhanced weight loss during treatment and attenuated weight regain following treatment withdrawal in obese mice. In addition, GB102 was associated with preservation of muscle strength, which is often reduced during pharmacologically induced weight loss. These findings suggest that GB102 may complement GLP-1RA-induced metabolic effects in this preclinical model.

The potential interaction between GB102 and GLP-1RA therapy may be interpreted in the context of complementary mechanisms. Whereas GLP-1RAs primarily act through appetite suppression, delayed gastric emptying, and insulinotropic effects, GB102 is associated with metabolic features linked to energy expenditure and amino acid metabolism. The combination of these distinct physiological domains may contribute to the improved outcomes observed in the present study. However, the precise mechanisms underlying this interaction were not directly investigated and remain to be elucidated.

This study has several limitations that should be acknowledged. First, this study was conducted exclusively in a mouse model, and therefore the translational relevance to human physiology remains uncertain. Second, although metabolomic profiling identified distinct microbial metabolites, corresponding host metabolite levels were not measured, limiting the ability to directly link microbial metabolism to systemic effects. Third, mechanistic pathways, including thermogenesis, nitric oxide signaling, and polyamine metabolism, were not directly assessed at the molecular level. Finally, the sample sizes were determined based on ethical considerations and standard practice for preclinical studies, but larger cohorts may provide additional statistical robustness.

## 5. Conclusions

In summary, we found that GB102 is associated with a distinct metabolic profile and multiple beneficial metabolic outcomes in a preclinical model of obesity. The combination of increased energy expenditure, preservation of muscle-related parameters, and attenuation of post-treatment weight regain in the context of GLP-1RA therapy highlights the potential for microbiota-based approaches to be used as adjunctive strategies. However, these findings are based on a preclinical model; therefore, further studies are warranted to clarify the underlying mechanisms and assess their relevance in a clinical setting.

## Data Availability

The original contributions presented in this study are included in the article and Supplementary Materials. Further inquiries can be directed to the corresponding author.

## Supplementary Materials

The following supporting information can be downloaded at: https://www.mdpi.com/article/10.3390/nu18071050/s1, Figure S1: Effects of GB102 on circulating metabolic hormones and adipokines in HFD-fed mice; Figure S2: Effects of GB102 on immune cell composition in eWAT; Table S1: Quantitative estimation of target metabolites from GB102, GB104, and GB201.

## Abbreviations

The following abbreviations are used in this manuscript:

AUC	Area under the curve
BAT	Brown adipose tissue
CE-TOFMS	Capillary electrophoresis time-of-flight mass spectrometry
CLAMS	Comprehensive Lab Animal Monitoring System
eWAT	Epididymal white adipose tissue
GIP	Glucose-dependent insulinotropic polypeptide
GLP-1RAs	Glucagon-like peptide-1 receptor agonists
GTT	Glucose tolerance test
H&E	Hematoxylin and eosin
HFD	High-fat diet
HMT	Human Metabolome Technologies
IACUC	Institutional Animal Care and Use Committee
ITT	Insulin tolerance test
MRS	Man–Rogosa–Sharpe
MSTFA	N-methyl-N-trimethylsilyl trifluoroacetamide
NCD	Normal chow diet
NEAAs	Non-essential amino acids
NO	Nitric oxide
PAI-1	Plasminogen activator inhibitor-1
PBS	Phosphate-buffered saline
TD-NMR	Time-domain nuclear magnetic resonance
Treg	Regulatory T cell
VCO_2_	Carbon dioxide production
VO_2_	Oxygen consumption
