# Supplementary material for: Energy-Metabolism-Enhancing Probiotics Enhance the Therapeutic Response to a Glucagon-like Peptide-1 Receptor Agonist
Source: Nutrients. 2026 Mar 26;18(7):1050. doi: 10.3390/nu18071050 (PMC13074448; doi:10.3390/nu18071050)
Supplement: Supplementary file 1 [file nutrients-18-01050-s001.zip › nutrients-4198894_Supplementary figures.docx]

**Supplementary Figures**

**
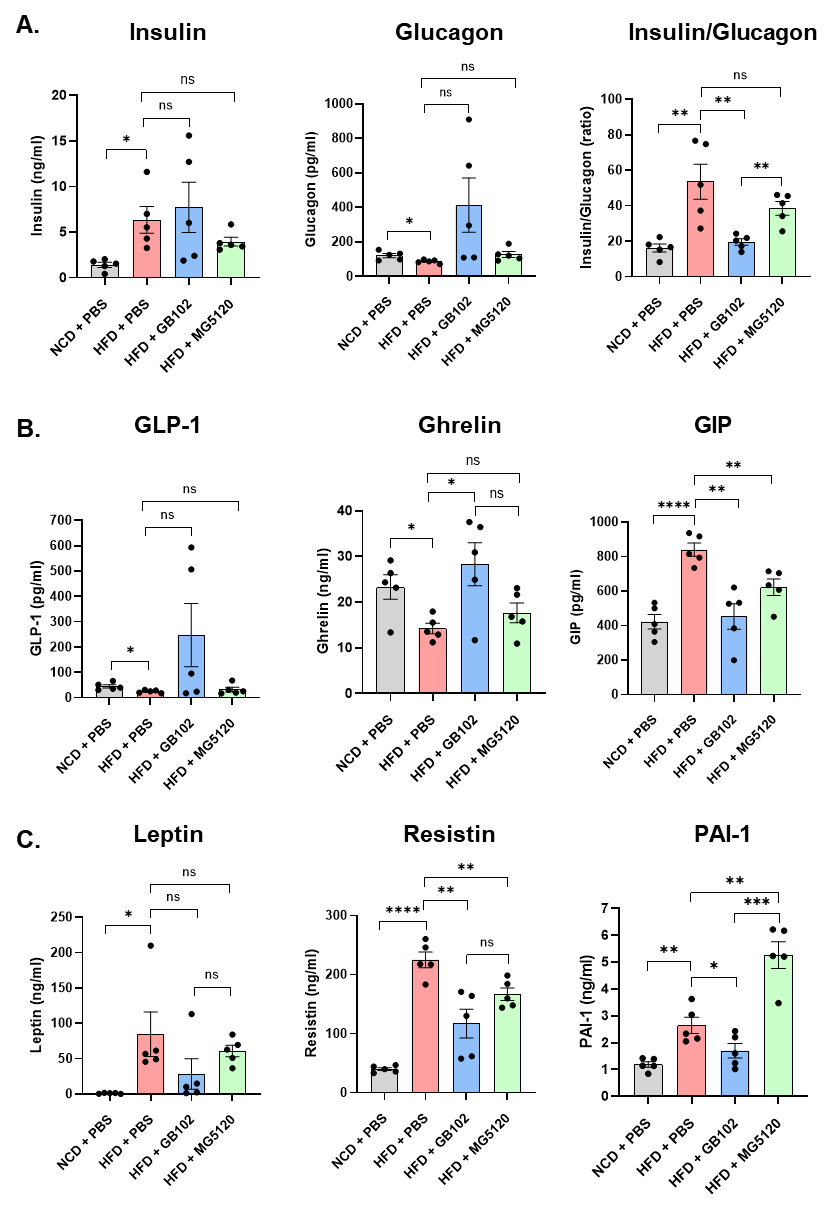
**

**Supplementary Figure S1.** Effects of GB102 on circulating metabolic hormones and adipokines in HFD-fed mice**.** (A-C) Serum levels of metabolic hormone measured after 14 weeks of GB102 or MG5120 administration (5 × 10⁹ CFU/head/day). (A) Glucose-regulating hormones. (B) Gut-derived hormones. (C) Adipose tissue-derived hormones and adipokines. Data are presented as mean ± SEM (n = 5 per group). **P* < 0.05; ***P* < 0.01; ****P* < 0.001; *****P* < 0.0001; ns, not significant.


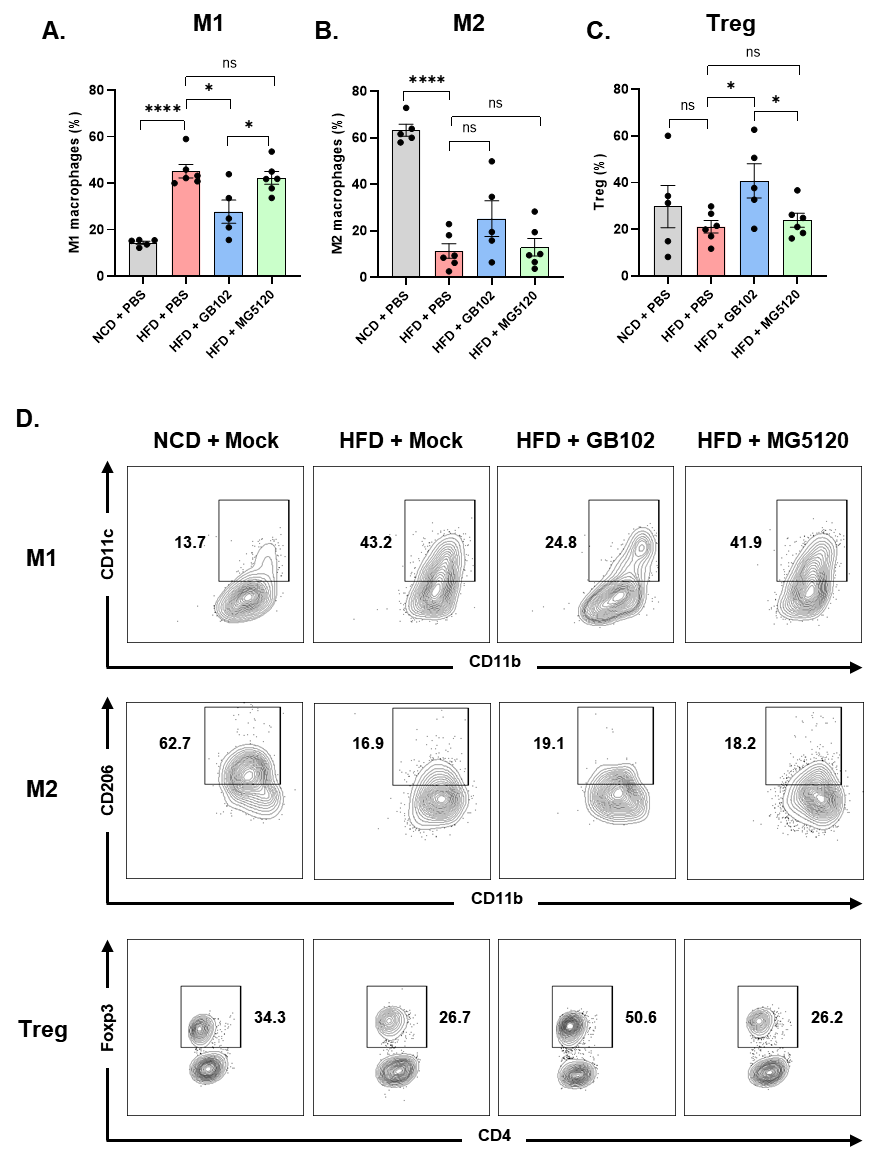


**Supplementary Figure S2.** Effects of GB102 on immune cell composition in eWAT. (A-C) Flow cytometric analysis of immune cell subsets in eWAT after 14 weeks of GB102 or MG5120 administration (5 × 10⁹ CFU/head/day). M1 and M2 macrophages were quantified after gating on MCHII^+^F4/80^+^ cells, and regulatory T cells (Treg) were analyzed after gating on TCRβ^+^CD4^+^ cells. (A) M1 macrophages. (B) M2 macrophages. (C) Tregs. (D) Representative flow cytometry plots. Data are presented as mean ± SEM (n = 5-6 per group). **P* < 0.05; *****P* < 0.0001; ns, not significant.
